# Smoking and Risk of Kidney Failure in the Singapore Chinese Health Study

**DOI:** 10.1371/journal.pone.0062962

**Published:** 2013-05-09

**Authors:** Aizhen Jin, Woon-Puay Koh, Khuan Yew Chow, Jian-Min Yuan, Tazeen Hasan Jafar

**Affiliations:** 1 National Registry of Diseases Office, Health Promotion Board, Singapore, Singapore; 2 Office of Clinical Sciences, Duke-NUS Graduate Medical School, Singapore, Singapore; 3 Department of Epidemiology, Saw Swee Hock School of Public Health, National University of Singapore, Singapore, Singapore; 4 Division of Cancer Control and Population Sciences, University of Pittsburgh Cancer Institute, Pittsburgh, Pennsylvania, United States of America; 5 Department of Epidemiology, Graduate School of Public Health, University of Pittsburgh, Pittsburgh, Pennsylvania, United States of America; 6 Laboratory of Cardiovascular and Renal Risk Reduction, Health Services & Systems Research, Duke-NUS Graduate Medical School, Singapore, Singapore; Mario Negri Institute for Pharmacological Research and Azienda Ospedaliera Ospedali Riuniti di Bergamo, Italy

## Abstract

**Background:**

The relationship between smoking and risk of kidney failure, especially in people of Chinese origin, is not clear. We analyzed data from the Singapore Chinese Health Study to investigate whether smoking increases the risk of kidney failure.

**Methods:**

The Singapore Chinese Health Study is a population-based cohort of 63,257 Chinese adults enrolled between 1993 and 1998. Information on smoking status was collected at baseline. Incidence of kidney failure was identified via record linkage with the nationwide Singapore Renal Registry until 2008. Kidney failure was defined by one of the following: 1) serum creatinine level of more than or equal to 500 µmol/l (5.7 mg/dl), 2) estimated glomerular filtration rate of less than 15 ml/min/1.73 m^2^, 3) undergoing hemodialysis or peritoneal dialysis, 4) undergone kidney transplantation. Cox proportional hazard regression analysis was performed for the outcome of kidney failure after adjusting for age, education, dialect, herbal medications, body mass index, sex, physician-diagnosed hypertension and diabetes mellitus.

**Results:**

The mean age of subjects was 55.6 years at baseline, and 44% were men. Overall 30.6% were ever smokers (current or former) at baseline. A total of 674 incident cases of kidney failure occurred during a median follow-up of 13.3 years. Among men, smokers had a significant increase in the adjusted risk of kidney failure [hazard ratio (HR): 1.29; 95% CI: 1.02–1.64] compared to never smokers. There was a strong dose-dependent association between number of years of smoking and kidney failure, (p for trend = 0.011). The risk decreased with prolonged cessation (quitting ≥10 years since baseline). The number of women smokers was too few for conclusive relationship.

**Limitation:**

Information on baseline kidney function was not available.

**Conclusions:**

Cigarette smoking is associated with increased risk of kidney failure among Chinese men. The risk appears to be dose- and duration-dependent and modifiable after long duration of cessation.

## Introduction

Kidney failure is a rising public health problem globally with high morbidity and reduced quality of life. [1]–[3]. The rapid increase in number of people with kidney failure, including those requiring or nearing the need for expensive renal replacement therapy, is in part due to the aging populations coupled with prolonged exposure to risk factors for kidney failure. [4] Smoking is recognized as one of the leading causes of preventable deaths globally predominantly from cardiovascular disease and cancer. [5] Evidence from European origin population indicates that smoking may be associated with progression of chronic kidney disease (CKD), especially in men. [6] However, there is scarcity of evidence on the relationship of smoking with kidney failure in non-European origin populations. Moreover, it is not known whether any potential relationship between smoking and kidney failure is dose-dependent, and, if so, is it modifiable. Asian populations have been shown to have greater susceptibility to CKD than European counterparts by virtue of a greater burden of many key risk factors, and there is evidence that CKD progresses to kidney failure faster in Asians. [7] Furthermore, the burden of kidney failure is increasing rapidly in many Asian countries where implementation of tobacco control policies has been challenging. [4], [8] However, it is unclear how smoking plays a role in the onset of kidney failure in Asians.

A clear understanding of the nature of association between smoking and kidney failure would be important for enhancing the understanding the epidemiology of CKD and for boosting smoking prevention and cessation efforts, especially among individuals at high risk for kidney failure in Asia.

We therefore analyzed data from the Singapore Chinese Health Study linked with the Singapore Renal Registry which maintains a record of all patients with kidney failure, treated with renal replacement therapy or managed conservatively, with the following objectives: 1) To determine the association between cigarette smoking status at baseline (former or current) with risk of kidney failure; 2) to assess whether there is a dose-dependent relationship between smoking intensity and duration and kidney failure risk, and 3) to assess whether quitting smoking reduces the risk of kidney failure.

We hypothesized that smoking would increase the risk of kidney failure; and that this risk would be dose dependent and modifiable with smoking cessation in men of Chinese origin.

## Methods

### Study Population

The Singapore Chinese Health Study is a population-based prospective cohort established between April 1993 and December 1998 through the recruitment of 63,257 Chinese men (n = 27,959) and women (n = 35,298), who were aged 45–74 years, and residing in public housing estates, where 86% of Singapore resided at that time. All participants belonged to one of the two major dialect groups, Hokkien or Cantonese, who originated from two contiguous prefectures in southern China. At recruitment, after obtaining informed consent, each study subject was interviewed in person by a trained interviewer using a structured questionnaire, which focused on history of tobacco and alcohol use, current diet, current physical activity, menstrual and reproductive history in women, medical history, and family history of cancer. Current diet was assessed using a validated 165-item, semi-quantitative food frequency questionnaire. [9] The Institutional Review Board at the National University of Singapore approved this study. Written informed consent was obtained from all participants.

### Exposure Assessment

For cigarette smoking, the study population was divided into never, former and current smokers based on their choice of three possible responses to the following question, “Have you ever smoked at least one cigarette a day for 1 year or longer”. Subjects who answered “no” were classified as “never-smokers”, those who answered “yes, but I quit smoking” were classified as “former smokers”, and those who answered “yes, and I currently smoke” were classified as “current smokers”. Ever smokers (former or current) were then asked about age at smoking initiation (four categories: less than 15, 15–19, 20–29, and 30 years or older); number of cigarettes smoked per day (six categories: 6 or less, 7–12, 13–22, 23–32, 33–42, and 43 or more); and duration of smoking (five categories; less than 10, 10–19, 20–29, 30–39, 40 or more years). Former smokers were also asked how long they had quit smoking (seven categories; less than 1 year, 1–2 years, 3–4 years, 5–9 years, 10–14 years, 15–19 years, 20 or more years).

### Case Ascertainment

Identification of incident kidney failure and deaths among cohort members was accomplished by record linkage analysis of the cohort database with the population-based Singapore Renal Registry. The nationwide renal registry has been in place since 1993 and has been shown to be comprehensive in its recording of kidney failure cases since 1999. [10] The registry defines kidney failure by at least one of these criteria: 1) serum creatinine level of more than or equal to 500 µmol/l (5.7 mg/dl) 2) estimated glomerular filtration rate (GFR) of less than 15 ml/min/1.73 m^2^ (based on either the Modification of Diet in Renal Disease (MDRD) Study equation, Cockcroft Gault equation, or 24 hour creatinine clearance) 3) undergoing hemodialysis or peritoneal dialysis, 4) had undergone kidney transplant. All cases are then classified by disease etiology based on review of medical records. As of April 2008, only 27 cases were known to be lost to follow-up due to migration out of Singapore. As of 31 Dec 2008, 94 participants had kidney failure diagnosed before recruitment and were excluded from the analysis. A total of 674 incident kidney failure cases were identified from the remaining 62,583 participants included in the analyses. Among them, 56.9% had diabetic nephropathy, 15.7% primary glomerulonephritis and 12.7% nephrosclerosis.

### Statistical Analysis

Since the development of kidney failure follows a chronic process over time, we examined baseline characteristics of the entire cohort by duration of smoking for men and women. For each study subject, person-years were counted from the date of baseline interview to the date of kidney failure diagnosis, the date of death, date of last contact (for the few subjects who migrated out of Singapore) or December 31, 2008, whichever occurred first. The incidence rate of kidney failure was computed and 95% CI were generated using Poisson distribution.

Univariable and multivariable regression models were constructed and Cox proportional hazards regression analysis was performed to examine the associations between cigarette smoking and risk of developing kidney failure. Deaths from competing causes (ie non kidney failure deaths) were censored in the time to event analysis for the primary outcome of kidney failure. We first examined the association of current and former smokers, and number of cigarettes smoked with kidney failure in the entire cohort. The number of cigarettes smoked was grouped into 3 categories of 12 or less per day, 13–22/day, or 23 or more per day. Since studies in other populations have provided sex-specific estimates [6], our subsequent main analyses were stratified for men and women. To increase power of association with smoking status, we grouped former and current smokers as “ever” smokers. The duration of smoking (<19 years; 19–39 years, 40 years or more) and time since quitting (quit for 1 year or less, quit >1–9 years, and quit for 10 years or more) were regrouped into 3 categories each per distribution of data. The associations were measured by hazard ratios (HRs) and their corresponding 95% confidence intervals (CIs) and p values (two-sided).

In the analysis considering smoking and kidney failure, the model included the following baseline covariates: age, body mass index, dialect (Hokkein, Cantonese), education level, history of physician diagnosed hypertension, diabetes, known heart disease or stroke, alcohol use and intake of ginseng. These factors are known to be associated with kidney failure in other studies. [11], [12] For the analysis by duration of smoking and smoking cessation, we performed tests for trend by entering ordinal categorical variables as continuous variables in the Cox regression models. We also tested for interaction between age and duration of smoking, number of cigarettes smoked with age at initiation of smoking. We performed analyses for the whole population and also for men and women separately. Sensitivity analysis were performed after retaining the original categories of smoking status as in the questionnaire for years of smoking (five categories) and quit smoking (seven categories), and after excluding kidney failure cases occurred within first 5 years post-enrollment, to reduce the impact of outcome on the change of smoking behavior, as above.

All regression analyses were conducted using SAS statistical software version 9.2 (SAS institute, Cary, NC). Two-sided *P*s <0.05 were considered statistically significant.

## Results

Of the 63163 subjects, 44.1% were men. Among men, 41.9% were never smokers, and 8.3%, 30.3%, and 19.6% smoked for <19, 19–39, and 40 years or more, respectively. Among women, 91.1% were never smokers, and 1.5%, 3.6%, and 3.6% smoked for <19, 19–39, and 40 years or more. Table 1 shows baseline characteristics of the participants by duration of smoking. Men who smoked were more likely to be less educated, consume alcohol, have diabetes, heart disease and stroke, and these associations were stronger with longer duration of smoking. During a median follow-up of 13.3 year, and 774,434 person years, 674 (1%) developed incident kidney failure with an overall crude incidence rate of 0.87 (0.81–0.94) per 1000 person years (Table 2), and age-standardized incidence rate of 0.83 per 1000 person years.

**Table 1:** Baseline characteristic of individuals by smoking status in Singapore Chinese Health Study.

	N = 63163	Men N = 27907					Women N = 35256				
		Men ever smokers(years of smoking)					Women ever smokers(years of smoking)				
		Never smokerN = 11706	> = 40 yearsN = 5491	20–39 yearsN = 8467	< = 19 yearsN = 2243	P for trend	Never smokerN = 32153	> = 40 yearsN = 1290	20–39 yearsN = 1274	< = 19 yearsN = 539	P for trend
**Age at recruitment (mean, SD)**	56.5 (8.0)	55.38 (7.8)	63.38 (6.0)	54.61 (7.1)	55.37 (7.9)	<0.0001	55.83 (7.9)	65.07 (5.6)	59.10 (7.2)	58.77 (8.2)	<0.0001
**Education %**											
** No formal education**	17308 (27.4)	877 (7.5)	1197 (21.8)	827 (9.8)	156 (7.0)	<0.0001	12341 (38.4)	897 (69.5)	740 (58.1)	273 (50.7)	<0.0001
** Primary**	28001 (44.3)	5051 (43.1)	3433 (62.5)	4698 (55.5)	1097 (48.9)		12725 (39.6)	345 (26.7)	444 (34.9)	208 (38.5)	
** Second &above**	17854 (28.3)	5778 (49.4)	861 (15.7)	2942 (34.7)	990 (44.1)		7087 (22.0)	48 (3.7)	90 (7.0)	58 (10.8)	
**Dialect %**											
** Cantonese**	29239 (46.3)	5756 (49.2)	2039 (37.1)	3414 (40.3)	1111 (49.5)	<0.0001	15444 (48.0)	611 (47.4)	569 (44.7)	295 (54.7)	0.0004
** Hokkein**	33924 (53.7)	5950 (50.8)	3452 (62.9)	5053 (59.7)	1132 (50.5)		16709 (52.0)	679 (52.6)	705 (55.3)	244 (45.3)	
**History of hypertension %**	14985 (23.7)	3062 (26.2)	1159 (21.1)	1667 (19.7)	633 (28.2)	<0.0001	7754 (24.1)	271 (21.0)	301 (23.6)	138 (25.6)	0.073
**History of diabetes %**	5673 (9.0)	1005 (8.6)	510 (9.3)	686 (8.1)	215 (9.6)	0.015	2850 (8.9)	172 (13.3)	171 (13.4)	64 (11.9)	0.641
**History of heart disease%**	2589 (4.1)	449 (3.8)	393 (7.2)	423 (5.0)	99 (4.4)	<0.0001	1028 (3.2)	94 (7.3)	73 (5.7)	30 (5.6)	0.194
**History of stroke %**	947 (1.5)	176 (1.5)	164 (3.0)	132 (1.6)	35 (1.6)	<0.0001	384 (1.2)	29 (2.3)	21 (1.7)	6 (1.1)	0.216
**Alcohol**											
** Never**	55853 (88.4)	9848 (84.1)	4281 (77.9)	6255 (73.9)	1771 (78.9)	<0.0001	30831 (95.9)	1197 (92.8)	1171 (91.9)	499 (92.6)	0.421
** Weekly**	5105 (8.1)	1486 (12.7)	690 (12.6)	1423 (16.8)	354 (15.8)		1017 (3.1)	47 (3.6)	65 (5.1)	23 (4.3)	
** Daily**	2205 (3.5)	372 (3.2)	520 (9.5)	789 (9.3)	118 (5.3)		305 (1.0)	46 (3.6)	38 (3.0)	17 (3.2)	
**BMI (mean, SD)**	23.1 (3.3)	23.3 (3.2)	22.2 (3.1)	22.9 (3.2)	23.5 (3.2)	<0.0001	23.3 (3.3)	22.6 (3.8)	23.0 (3.5)	23.2 (3.5)	0.0006
**Ginseng**											
** Never**	52582 (83.3)	10009 (85.5)	4755 (86.6)	7258 (85.7)	1866 (83.2)	0.006	26142 (81.3)	1082 (83.9)	1044 (82.0)	426 (79.0)	0.124
**1–3/month**	2884 (4.6)	442 (3.8)	233 (4.2)	339 (4.0)	108 (4.8)		1587 (4.9)	73 (5.7)	71 (5.5)	31 (5.8)	
**1–3/week**	6039 (9.5)	963 (8.2)	395 (7.2)	686 (8.1)	214 (9.5)		3482 (10.8)	103 (8.0)	131 (10.3)	65 (12.1)	
**4–6/week and more**	1658 (2.6)	292 (2.5)	108 (2.0)	184 (2.2)	55 (2.5)		942 (2.9)	32 (2.4)	28 (2.2)	17 (3.1)	

**Table 2:** Incidence of Kidney Failure by Smoking Status in Singapore Chinese Health Study.

	All	Men ever smoker (years of smoking)				Women ever smoker(years of smoking)			
		Never smoker	> = 40 years	20–39 years	< = 19 years	Never smoker	> = 40 years	20–39 years	< = 19 years
**Persons**	63163	11706	5491	8467	2243	32153	1290	1274	539
**Person-years**	774434.1	144629.2	56769.7	102753.7	27097.8	407116.6	14116.4	15498.4	6452.3
**Median of follow-up**	13.3	13.3	11.3	13.2	12.6	13.6	11.9	13.4	12.7
**Kidney Failure (N)**	674	116	72	89	27	327	15	21	7
**Incidence rate per 1000 py**	0.870 (0.805–0.936)	0.802 (0.656–0.948)	1.268 (0.975–1.561)	0.866 (0.686–1.046)	0.996 (0.621–1.372)	0.803 (0.716–0.890)	1.063 (0.525–1.600)	1.355 (0.775–1.934)	1.085 (0.281–1.889)

The hazard rates (95% CI) of kidney failure by smoking status are shown in Tables 3, 4, and 5. In the entire cohort, smoking was associated with increased risk of kidney failure [hazard ratio (HR), 1.42; 95% confidence interval (CI): 1.07–1.87] for former smokers, and [HR (95% CI): 1.28 (0.98–1.65)] for current smokers: [p for trend = 0.04], adjusted for age, body mass index, dialect, education level, history of physician diagnosed hypertension, diabetes, known heart disease or stroke, alcohol use and intake of ginseng compared to never smokers. The risk increased with increased number of cigarettes (p for trend = 0.03) (Table 3). Among men, ever smokers had a significant increase in the risk of kidney failure [adjusted HR (95% CI): (1.29 1.02–1.64)], compared to never smokers (Table 4). There was a strong dose-dependent association between duration of smoking and kidney failure (p for trend = 0.011). Men who had smoked for 40 years or more at baseline had significantly increased adjusted risk of kidney failure compared to men who never smoked [HR (95% CI): 1.56 (1.23–2.21)]. These relationships seemed to be similar in women with unadjusted hazard rate of kidney failure was [HR (95% CI)] 1.51 (1.10–2.08)] in ever versus never smokers, and with increasing duration of smoking (p for trend 0.01), albeit the adjusted risks were not statistically significant. As shown in Table 5, the risk of kidney failure remained significantly elevated even 1–9 years after smoking cessation. However, the adjusted HR decreased to 1.02 at 10 years or greater after quitting. A similar pattern was observed in women, albeit no significant association was observed in the adjusted model due to small case numbers among women.

**Table 3:** Risk of Kidney Failure by Smoking Status in Singapore Chinese Cohort Study.

Smoking status	Number Kidney Failure	*Adjusted HR (95% CI)
**Never smoker (n = 43859)**	443	1.00
**Former smoker (n = 6976)**	108	1.42 (1.07–1.87)
**Current smoker (n = 12327)**	122	1.28 (0.98–1.65)
**P trend**		0.04
**Never smoker (n = 43859)**	443	1.00
**Ever smoked < = 12 cigarettes/day (n = 7693)**	95	1.27 (1.00–1.61)
**Ever smoked 13–22 cigarettes/day (7331)**	76	1.15 (0.88–1.50)
**Ever smoked > = 23 cigarettes/day (n = 4280)**	60	1.37 (1.01–1.84)
**P trend**		0.03

* Model adjusted for age, body mass index, dialect (Hokkein, Cantonese), education level, history of physician diagnosed hypertension, diabetes, known heart disease or stroke, alcohol use and intake of ginseng.

**Table 4:** Unadjusted & Adjusted Risk of Kidney Failure in Chinese Men and Women by Smoking Status.

	Men		Women	
Smoking Status	Unadjusted Hazard Ratio (95% CI)	*Multivariable Adjusted HazardRatio (95% CI)	Unadjusted Hazard Ratio (95% CI)	*Multivariable Adjusted Hazard Ratio (95% CI)
**Never vs**	1.00	1.00	1.00	1.00
**Ever**	1.28 (1.01–1.61)	1.29 (1.02–1.64)	1.51 (1.10–2.08)	1.18 (0.85–1.62)
**Never Smoker vs**	1.00	1.00	1.00	1.00
**Duration of smoking <19 years**	1.26 (0.83–1.91)	1.17 (0.77–1.78)	1.37 (0.65–2.90)	1.19 (0.56–2.51)
**Duration of smoking 20–39 years**	1.08 (0.82–1.43)	1.19 (0.90–1.58)	1.70 (1.09–2.64)	1.28 (0.82–2.00)
**Duration of smoking >40 years**	1.65 (1.23–2.21)	1.56 (1.13–2.14)	1.37 (0.81–2.29)	1.04 (0.62–1.76)
**P trend**	0.007	0.011	0.019	0.435

Never smoker was defined as those who have not ever smoked at least one cigarette a day for 1 year or longer.

Ever smoker was defined as those who have ever smoked at least one cigarette a day for 1 year or longer.

* Model adjusted for age, body mass index, dialect (Hokkein, Cantonese), education level, history of physician diagnosed hypertension, diabetes, known heart disease or stroke, alcohol use and intake of ginseng.

**Table 5:** Association of smoking cessation with Kidney Failure in Singapore Chinese Health Study.

Smoking Status	N	Person years	Number Kidney Failure	Unadjusted Hazard Ratio (95% CI)	Multivariable **Adjusted Hazard Ratio (95% CI)
**Men**					
**Never Smoker vs.**	11705	144623	115	1.00	1.00
**Quit smoking <1 year**		20633	19	1.18 (0.73–1.92)	1.26 (0.77–2.08)
**Quit smoking 1–9 years**	1856	26472	45	2.17 (1.54–3.06)	1.83 (1.29–2.60)
**Quit smoking >10 years**	2333	37880	40	1.36 (0.95–1.95)	1.02 (0.71–1.47)
**P trend**	3301			0.002	0.22
**Women**					
**Never Smoker vs.**	32152	407106	326	1.00	1.00
**Quit smoking <1 year**	234	2636	3	1.45 (0.46–4.50)	1.25 (0.40–3.91)
**Quit smoking 1–9 years**	353	3846	9	2.15 (1.55–5.82)	1.61 (0.83–3.14)
**Quit smoking >10 years**	445	4929	5	1.33 (0.55–3.20)	0.79 (0.32–1.92)
**P trend**				0.003	0.72

* Model adjusted for age, body mass index, dialect (Hokkein, Cantonese), education level, history of physician diagnosed hypertension, diabetes, known heart disease or stroke, alcohol use and intake of ginseng.

Finally, the sensitivity analysis revealed consistent results with respect to the main associations as well as the dose dependency of the relationship between smoking and kidney failure, and the threshold of 10 years since quitting for the risk to reduce substantially.

## Discussion

Our in-depth analysis data on 63,163 subjects followed over a median of 13.3 years with 674 incident kidney failure in the Singapore Chinese Health Study showed an increased risk of kidney failure that correlated with duration of smoking in men. The adjusted risk estimate decreased in men who quit smoking after prolonged cessation, although it was still significantly elevated in those who had quit for less than 10 years. The direction of relationship between smoking and kidney failure were similar in women however the numbers were too small to be conclusive.

Our findings in Chinese men are consistent with observations of the Multiple Risk Factor Intervention Trial (MRFIT) which studied 332,544 men and documented that smoking was significantly associated with an increased risk for kidney failure [11]. Our study is novel in demonstrating the dose-relationship between smoking and kidney failure in non-European origin population (unadjusted p trend 0.007, adjusted p trend = 0.01). This association has been previously documented between number of cigarettes smoked and albuminuria and decline in kidney function, however not with the hard outcome of kidney failure. [13] Our results are also among the first to suggest that prolonged smoking cessation >10 years since baseline may reduce this risk back to the level of a non-smoker (HR = 1.02 (0.71–1.47) (Table 5, column 4, row 1). This finding is consistent with those recently reported in Europeans in the HUNT Study. [6] However, we did not find a clear graded trend with increasing years of smoking cessation and kidney failure (adjusted trend p = 0.22) indicating the possibility of a threshold duration. Overall, these findings support the notion of shared risk factors for CVD and kidney disease. [14] However, they also contrast with the effects of smoking on CVD which have been shown to reduce immediately within 2–3 years of smoking cessation. [15]–[17]While we are unable to evaluate the pathophysiology of the association, smoking is known to affect endothelial cell function and nitric oxide production leading to vasoconstriction and vascular damage. [18] It is highly conceivable that these mechanisms contribute, at least in part, to the progressive renal damage associated with smoking.

It is also important to underscore that the incidence of kidney failure (674 cases in a cohort of 63,163 over 13.4 years (Table 2)) is higher in our study than previously reported in European origin populations such as the MRFIT (814 individuals developed kidney failure among a cohort of 332,544 men over 16 years), HUNT, and other studies in European-origin populations, and consistent with high incidence of kidney failure reported in the Southeast Asian population in Taiwan. [19], [20] It remains to be studied whether this variation is due to a greater prevalence, faster progression of CKD, or both, or perhaps improved survival from competing causes in the Chinese population with a different sociodemographic profile than their Western counterparts. [21] Nevertheless our findings indicate that smoking contributes to the burden of kidney failure.

The major strengths of our findings are the large, representative, population based cohort with well documented information on smoking status linked with a robust renal registry, and all results regarding key relationships with smoking status (dose response with regard to duration and intensity) were consistent in direction in sensitivity analysis in the entire cohort. A limitation of the study is the lack of information on baseline kidney function including serum creatinine or albuminuria, and non-kidney failure deaths were censored in the analysis. This precluded evaluation of the effect of smoking on other causes (especially cardiovascular deaths) in earlier stages on CKD. Clearly such a relationship will magnify the detrimental effect of smoking across the spectrum of CKD as smoking has been shown to predict CVD and cancer deaths in this cohort. [22] However, our main objective was to determine the relationship of smoking with kidney failure which we were able to demonstrate. Second, data on reno-protective antihypertensive medications including blockers of renin-angiotensin, and reduction in blood pressure or albuminuria during follow-up were not available. [23] We were therefore not able to determine any potential interaction between these factors with smoking status on kidney failure. However, our multivariable model accounted for hypertension and diabetes at baseline, both leading causes of kidney failure, as well as predictors of progression. [11] A third limitation is the possibility that the observed relationship between smoking and kidney failure is confounded by unmeasured social determinant (household income, neighborhood poverty index) including access to healthcare known to be associated with kidney failure. [24]–[27] Nevertheless, the reduction in risk of kidney failure with smoking cessation is highly suggestive of an independent predictive and causal nature of the association. Finally the number of women smokers was too low to detect any significant associations, and it is possible that clinically meaningful interactions (eg with sociodemographic factors) remained undetected. Future research on larger pooled data may provide further valuable insights in potential heterogeneity of the relationship of smoking with kidney failure in these subgroups.

In summary, our results on analysis of the Singapore Chinese cohort indicate a strong predictive association of ever smoking with kidney failure, which increases with the duration of smoking. This risk reduces back to the level of a non-smoker only after very prolonged duration of smoking cessation. Our findings have tremendous implications for public policy globally, especially in Southeast Asian countries and China. The number of people with kidney failure in this region is increasing rapidly with aging populations. Despite the global initiative of the World Health Assembly and ratification of the Framework Convention on Tobacco Control (FCTC), over 1 billion people smoke globally, a substantial proportion of the population of Chinese origin is exposed. [28], [29] Our results suggest that if left unchecked, a further escalation in the number of people with kidney failure is expected. Immediate action is therefore needed for intensifying tobacco control efforts through legislative action, taxation, public education, collaborative partnership to reduce the supply and demand of tobacco and provision of smoking cessation services, and targeted intervention for individuals at high risk of kidney failure.
